# Mated Progeny Production Is a Biomarker of Aging in *Caenorhabditis elegans*

**DOI:** 10.1534/g3.113.008664

**Published:** 2013-10-18

**Authors:** Christopher L. Pickett, Nicholas Dietrich, Junfang Chen, Chengjie Xiong, Kerry Kornfeld

**Affiliations:** * Department of Developmental, Biology Washington University School of Medicine, St. Louis, Missouri 63110; † Division of Biostatistics, Washington University School of Medicine, St. Louis, Missouri 63110

**Keywords:** *C. elegans*, aging, reproduction, longitudinal study, biomarker

## Abstract

The relationships between reproduction and aging are important for understanding the mechanisms of aging and evaluating evolutionary theories of aging. To investigate the effects of progeny production on reproductive and somatic aging, we conducted longitudinal studies of *Caenorhabditis elegans* hermaphrodites. For mated wild-type animals that were not sperm limited and survived past the end of the reproductive period, high levels of cross-progeny production were positively correlated with delayed reproductive and somatic aging. In this group of animals, individuals that generated more cross progeny also reproduced and lived longer than individuals that generated fewer cross progeny. These results indicate that progeny production does not accelerate reproductive or somatic aging. This longitudinal study demonstrated that cumulative cross progeny production through day four is an early-stage biomarker that is a positive predictor of longevity. Furthermore, in mated animals, high levels of early cross progeny production were positively correlated with high levels of late cross progeny production, indicating that early progeny production does not accelerate reproductive aging. The relationships between progeny production and aging were further evaluated by comparing self-fertile hermaphrodites that generated relatively few self progeny with mated hermaphrodites that generated many cross progeny. The timing of age-related somatic degeneration was similar in these groups, suggesting progeny production does not accelerate somatic aging. These studies rigorously define relationships between progeny production, reproductive aging, and somatic aging and identify new biomarkers of *C. elegans* aging. These results indicate that some mechanisms or pathways control age-related degeneration of both reproductive and somatic tissues in *C. elegans*.

Reproduction is a vital goal of all organisms and a central issue in research on aging. One important intersection between reproduction and aging concerns the mechanisms of age-related degeneration. In particular, are age-related degenerative changes in reproductive and somatic tissues controlled by similar or distinct mechanisms? Whereas a substantial number of genetic and environmental factors have been demonstrated to influence somatic aging and life span, fewer factors are known to influence reproductive aging. In female humans, reproductive aging is an important medical issue because the age-related decrease in oocyte quality results in increased birth defects and decreased fertility that culminates in reproductive cessation at menopause (te Velde and Pearson 2002; Hartge 2009). A second important intersection between reproduction and aging concerns how the production of progeny affects aging. The disposable soma theory proposes resources used for reproduction are not available for somatic maintenance so that reproductive activity accelerates age-related degeneration (Kirkwood 1977). The antagonistic pleiotropy theory proposes that mutations that increase early reproduction will increase fitness despite accelerating the decrease of late reproduction and shortening life span (Williams 1957). In contrast, multilevel selection theories propose that reproductive restraint can be an adaptive trait that stabilizes the population and prevents overexploitation of the environment (Wynne-Edwards 1962), and we have previously proposed that reproductive aging is one mechanism of reproductive restraint (Hughes *et al.* 2007). Experimental tests of the evolutionary theories of aging are critical to advance our understanding of the causes and mechanisms of reproductive and somatic aging.

The nematode *Caenorhabditis elegans* is an important model organism for studies of aging (Guarente and Kenyon 2000; Kenyon 2010). *C. elegans* hermaphrodites have a reproductive period of ~10 days and a postreproductive period of ~6 days for a total adult life span of ~16 days. Many age-related degenerative changes in reproductive and somatic function have been characterized (Collins *et al.* 2008; Pincus and Slack 2010). Sophisticated genetic approaches have resulted in the identification of many genes that modulate life span and/or reproductive span, providing insight into the genetic control of somatic and reproductive aging (Huang *et al.* 2004; Hughes *et al.* 2007; Andux and Ellis 2008; Luo *et al.* 2009; Kenyon 2010; Luo *et al.* 2010; Mendenhall *et al.* 2011). These studies have demonstrated important roles for, among others, insulin/insulin-like growth factor-1 signaling, mitochondrial function, chemosensory function, dietary intake, and autophagy in modulating adult life span (Kenyon 2010). *C. elegans* is also a useful model for testing evolutionary theories of aging due to the ability to maintain large populations, the variety of culture methods, and the wealth of bioinformatic tools (*C. elegans* Sequencing Consortium 1998; Stiernagle 2006; Gerstein *et al.* 2010). *C. elegans* is an androdioecious (male/hermaphrodite) species that evolved from gonochoristic (male/female) ancestors (Kiontke *et al.* 2004; Cutter *et al.* 2008; Morsci *et al.* 2011). There is growing interest in the natural history of *C. elegans*, but most information is derived from laboratory studies (Caswell-Chen *et al.* 2005; Felix and Braendle 2010). One caveat to using inbred laboratory strains for evolutionary experiments is that inbreeding often fixes rare, recessive, deleterious alleles resulting in the phenomena of inbreeding depression and heterosis (Rose and Charlesworth 1981a; Reznick 1985). However, inbred *C. elegans* wild-type (WT) strains do not suffer from these limitations because self-fertilization, the preferred method of maintaining *C. elegans*, tends to purge deleterious alleles (Johnson and Wood 1982; Lande and Schemske 1985; Johnson and Hutchinson 1993).

Here, we used *C. elegans* to investigate the relationships among progeny production, reproductive aging, and somatic aging. To determine the effect of reproduction on aging, we compared reproductive and somatic declines in genetically identical animals that produced different numbers of progeny due to differences in sperm availability (Ward and Carrel 1979; Hughes *et al.* 2007). Increased levels of progeny production did not accelerate somatic aging, indicating that reproductive activity does not cause somatic aging. Longitudinal studies were used to establish the relationships between different aging phenotypes in mated, WT hermaphrodites that were not sperm limited and survived past the end of the reproductive period (Huang *et al.* 2004). High levels of cross progeny production were positively correlated with extended longevity, and this relationship applied to total progeny production, cumulative progeny production through day 4, and progeny production on days 2, 4, and 5. These findings demonstrate that progeny production on day 2 is predictive of longevity, and they provide an independent line of evidence that progeny production does not accelerate somatic aging. These longitudinal studies also demonstrated that high levels of early cross progeny production were positively correlated with high levels of late cross progeny production, indicating that reproductive activity does not accelerate reproductive aging. These findings reveal novel biomarkers of *C. elegans* aging and indicate that some mechanisms promote the longevity of both reproductive and somatic function in this animal.

## Materials and Methods

### Nematode methods

*C. elegans* were maintained as described previously (Brenner 1974; Stiernagle 2006). Worms were cultured at 20° on 6-cm dishes with nematode growth medium and a lawn of *Escherichia coli* OP50. The WT strain (referred to as WT) was N2-Bristol (Brenner 1974), and the following mutations were used: *fer-6(hc6)* I (Ward *et al.* 1981), *lov-1(sy582)* II (Barr and Sternberg 1999), *fog-2(q71)* V (Clifford *et al.* 2000), and *him-5(e1490)* V (Hodgkin *et al.* 1979).

### Cross-sectional assays

One WT L4-stage hermaphrodite was placed on a dish with three WT L4 or young adult males (mated to WT), or 20 WT L4-stage hermaphrodites were placed on a dish (self-fertile). After 24 hr, self-fertile and mated hermaphrodites were transferred to fresh dishes. For mated hermaphrodites, progeny laid on day 2 were analyzed for the presence of males; the absence of male progeny indicated mating did not occur, and these hermaphrodites were excluded from the analysis. Self-fertile and mated hermaphrodites were transferred to fresh dishes every 2 d until the end of reproduction and then every 5 d.

### Longitudinal assays

One WT L4-stage hermaphrodite was placed on a dish with no males (self-fertile), three L4 or young adult WT males (mated to WT), three L4 or young adult *lov-1(sy582)*; *him-5(e1492)* males (mated to *lov-1*), or three L4 or young adult *fer-6(hc6)* males (mated to *fer-6*) (Stiernagle 2006). After 24 hr, the hermaphrodite was transferred to a fresh dish. Co-culture with males was limited to 24 hr to minimize the physical trauma caused by males (Gems and Riddle 1996; Hughes *et al.* 2007). Hermaphrodites were transferred to fresh dishes daily until egg laying ceased and then every 5 d until death. We defined the L4 stage as day 1. For mated hermaphrodites, animals that generated only self-progeny or switched from cross-progeny to self-progeny were excluded from the analysis (Ward and Carrel 1979; Hughes *et al.* 2007). For studies of *fog-2(q71)*, *fog-2(q71)* females were substituted for WT hermaphrodites.

A dissecting microscope was used to score the following phenotypes: (1) Progeny production was measured by manual counting of live progeny present on the surface or edge of the dish 2−4 d after the hermaphrodite was removed. (2) Reproductive span was determined by scoring the number of days a hermaphrodite generated live progeny. (3) Coordinated body movement span was determined by scoring the number of days a hermaphrodite exhibited continuous forward movement in response to vigorous dish tapping. Animals were observed for 10−30 sec. (4) Pharyngeal pumping span was determined by scoring the number of days an animal contracted its pharynx ≥25 times per min. Pharyngeal contractions were observed during a 10-sec interval. (5) Life span was determined by scoring the number of days an animal exhibited spontaneous movement or responded to prodding with a platinum wire (Huang *et al.* 2004; Hughes *et al.* 2007).

### Statistical analyses

Hermaphrodites or females that desiccated on the side of the dish, died from internally hatched progeny (matricidal hatching), extruded their intestines through their vulva (rupture), were sterile, or had a postreproductive span of <1 d were excluded from the final analysis (Huang *et al.* 2004; Evason *et al.* 2005, 2008; Hughes *et al.* 2007). Distribution of each measured variable was assessed by PROC UNIVARITE/SAS (SAS Institute, Inc. Cary, NC). When data deviated severely from normal distributions, logarithmic transformation was used to approximate the normality. Statistical comparisons between two worm groups were implemented using PROC MANOVA (SAS Institute, Inc. Cary, NC) and further confirmed by Wilcoxon’s nonparametric tests by PROC NPAR1WAY/SAS (SAS Institute, Inc.). Pearson correlation was used to assess the strength of association across different measures after log transformation of the data to approximate a normal distribution.

In five separate longitudinal trials, we analyzed a total of 72 WT hermaphrodites that were self-fertile and a total of 272 WT hermaphrodites mated to WT males. For WT self-fertile hermaphrodites, four animals died by matricidal hatching, one animal desiccated on the side of the dish, and 67 animals were included in the final data set. For those mated to WT males, 84 did not mate successfully, 101 animals died by matricidal hatching, and 21 animals desiccated on the side of the dish, ruptured, or had postreproductive spans <1 d, and 64 animals were included in the final data set.

For WT hermaphrodites mated to *lov-1(sy582)*; *him-5(e1490)* males, a total of 16 hermaphrodites were analyzed: 2 animals died by matricidal hatching, 1 animal desiccated on the side of the dish, and 13 animals were included in the final data set.

For WT hermaphrodites mated to *fer-6(hc6)* males, a total of 16 hermaphrodites were analyzed: 1 animal desiccated on the side of the dish, and 15 animals were included in the final data set.

For unmated *fog-2(q71)* females, a total of 96 animals were analyzed in two separate trials: 54 animals ruptured, 1 animal desiccated on the side of the dish, and 41 animals were included in the final data set. For *fog-2(q71)* females mated to WT males, 96 animals were analyzed in two separate trials: 3 animals desiccated on the side of the dish, 46 animals died by matricidal hatching, 2 animals ruptured, and 45 animals were included in the final data set.

Age-related matricidal hatching is caused by age-related degeneration of the egg-laying system prior to the end of progeny production (Pickett and Kornfeld 2013). The frequency of matricidal hatching varied in these experiments depending on the genotype and mating regimen. We previously reported an analysis of the animals in these experiments that died due to matricidal hatching (Pickett and Kornfeld 2013). To focus this study on animals that had complete documentation of reproductive effort and senescent changes, we excluded animals that died by matricidal hatching from the data analysis. Thus, conclusions of this study apply only to animals that do not display matricidal hatching. The supplemental data set displays the outcome of every animal that was analyzed.

## Results

### Increased progeny production caused by mating WT hermaphrodites did not accelerate somatic aging

To determine the effects of progeny production on somatic aging, we analyzed hermaphrodites with the identical genotype and different levels of progeny production. We manipulated progeny production by mating hermaphrodites to males, thereby increasing the availability of sperm and the level of progeny production. Exposing hermaphrodites to males has multiple effects. The desired effect is sperm transfer that promotes progeny production, but other effects include physical contact during copulation and transfer of seminal fluid. Previous studies examining the effects of male mating on hermaphrodite life span indicated that continual exposure to males reduces hermaphrodite life span by a mechanism independent of egg production but related to the stress of copulation (Van Voorhies 1992; Gems and Riddle 1996). We previously determined that 24−48 hr was the minimum period of time that a hermaphrodite could be exposed to males and still consistently receive sufficient sperm for the duration of the life span (Hughes *et al.* 2007). To minimize the stress of copulation and still achieve a high probability of effective sperm transfer, we exposed hermaphrodites to males for a minimal, 24-hr period.

To examine progeny production, somatic aging and reproductive aging in detail, we performed a longitudinal analysis that involved serial measurements of progeny production, body movement, pharyngeal pumping, and survival for each individual. Hermaphrodites that received no sperm or only a small amount of sperm during the 24-hr mating period were recognized by self-progeny production and excluded from the data analysis. To focus on animals that died as a result of aging, we excluded animals that died during the reproductive period due to internal hatching of progeny or died at any time due to accidental causes such as desiccation on the side of the dish; we only analyzed animals that survived past the end of the reproductive period as documented by at least one day of post-reproductive life span. The fate of every animal that started the experiment is shown in the Supporting Information, File S1. As expected, >99% of the eggs laid by mated and self-fertile animals hatched, and the progeny developed to adulthood (Ward and Carrel 1979; Hughes *et al.* 2007). Consistent with previous studies, self-fertile animals laid unfertilized oocytes and retained unfertilized oocytes in their germlines or uteri at the end of the reproductive period, whereas mated animals did not display these characteristics of sperm depletion (Ward and Carrel 1979; Hughes *et al.* 2007). These data indicate that mating maximizes the number of progeny an animal can generate.

**Figure 1:**
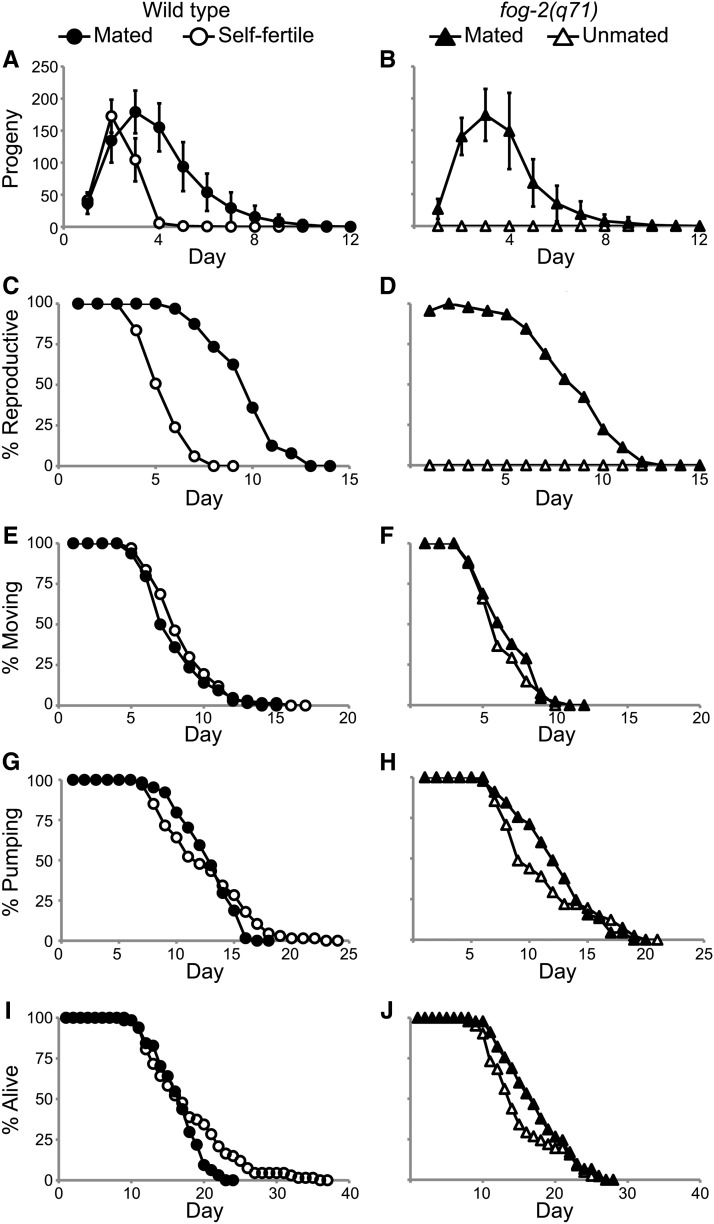
Increased progeny production in mated animals did not accelerate somatic aging. (A, B) Live progeny production, (C, D) reproductive span, (E, F) coordinated body movement span, (G, H) pharyngeal pumping span, and (I, J) life span were quantified for mated and self-fertile WT hermaphrodites and mated and unmated *fog-2(q71)* females analyzed using a longitudinal study design. The data in (A), (B), (I), and (J) were reported in Pickett and Kornfeld (2013).

Self-fertile, WT hermaphrodites generated 330 ± 40 self-progeny and displayed a reproductive span of 4.6 ± 0.9 days before reproduction ceased due to sperm depletion (Figure 1, A and C and Table 1B). After mating to WT males, WT hermaphrodites that were not sperm limited and survived past the end of the reproductive period displayed a brood size of 710 ± 180 progeny, a 115% increase (Figure 1A and Table 1B). Reproductive span increased to 8.8 ± 1.8 d, a 91% increase (Figure 1C and Table 1B). To analyze these data, we conducted four different statistical tests: analysis of variance (ANOVA), ANOVA after log transformation (log ANOVA), the Wilcoxon test, and the log-rank test (see *Materials and Methods*). ANOVA is a parametric test based upon the assumption that the data are sampled from a Gaussian distribution; a log transformation improves the fit of the data to a Gaussian distribution. The Wilcoxon and log-rank tests are nonparametric tests that are not based on an assumption about the distribution of the data. It is informative to analyze these data using several statistical tests because measurements such as brood size may have a different distribution in the population compared to measurements such as aging spans. Progeny production of mated WT animals was significantly greater than progeny production of self-fertile WT animals, and mated reproductive span was significantly longer than self-fertile reproductive span based on all four statistical tests (p_ANOVA_ < 0.001, p_log ANOVA_ < 0.001, p_Wilcoxon_ < 0.001, p_log-rank_< 0.001) (Table 1B).

**Table 1:** Summary statistics for reproduction and aging assays

	Mean	ANOVA*^a^*	Log-rank*^a^*	log ANOVA*^b^*	Wilcoxon*^b^*
A: Summary statistics for life span of WT animals, cross-sectional					
Life span*^c^*					
Mated	15.2 ± 3.8	0.1106	0.2386	0.0774	0.1366
Self-fertile	16.2 ± 3.3				
B: Summary statistics for WT animals, longitudinal					
Brood size*^c^*					
Mated	710 ± 180	<0.001	<0.001	<0.001	<0.001
Self-fertile	330 ± 40				
Reproductive span*^c^*					
Mated	8.8 ± 1.8	<0.001	<0.001	<0.001	<0.001
Self-fertile	4.6 ± 0.9				
Movement span*^c^*					
Mated	7.1 ± 2.1	0.1601	0.1991	0.1354	0.1184
Self-fertile	7.7 ± 2.2				
Pumping span*^c^*					
Mated	11.9 ± 2.4	0.6162	0.3481	0.2814	0.4549
Self-fertile	11.6 ± 3.8				
Life span*^c^*					
Mated	15.6 ± 3.3	0.0809	0.0137	0.2309	0.3967
Self-fertile	17.1 ± 5.8				
C: Summary statistics for *fog-2(q71)* animals, longitudinal					
Brood size*^c^*					
Mated	630 ± 180	N/A*^d^*	N/A*^d^*	N/A*^d^*	N/A*^d^*
Unmated	0 ± 0				
Reproductive span*^c^*					
Mated	7.7 ± 2.3	N/A*^d^*	N/A*^d^*	N/A*^d^*	N/A*^d^*
Unmated	0 ± 0				
Movement span*^c^*					
Mated	5.8 ± 1.9	0.3062	0.2701	0.3561	0.3381
Unmated	5.4 ± 1.7				
Pumping span*^c^*					
Mated	11.3 ± 3.3	0.1549	0.4617	0.0938	0.0722
Unmated	10.2 ± 3.9				
Life span*^c^*					
Mated	16.3 ± 4.6	0.0664	0.1787	0.0443	0.0359
Unmated	14.4 ± 4.9				

Summary statistics for self-fertile WT hermaphrodites and WT hermaphrodites mated to WT males and evaluated in a (A) cross-sectional study or (B) longitudinal study, and (C) unmated *fog-2(q71)* females and *fog-2(q71)* females mated to WT males evaluated in a longitudinal study. The number of animals analyzed was (A) mated = 54, self-fertile = 81, (B) mated = 64, self-fertile = 67, and (C) mated = 45, unmated = 41. The brood size and life spans of self-fertile and mated WT hermaphrodites analyzed longitudinally and mated *fog-2(q71)* females were reported in Pickett and Kornfeld (2013). ANOVA, analysis of variance; WT, wild type; N/A, not available.

a *P* values for univariate ANOVA or log-rank analysis.

b To approximate normal data distribution, we conducted ANOVA after log transformation of the data (log ANOVA) and the nonparametric Wilcoxon test.

c Brood size is measured as total progeny production. Spans are measured in days. All values are mean ± SD.

d ANOVA and Wilcoxon tests are not appropriate for these comparisons given the 0 values for unmated *fog-2(q71)* brood size and reproductive span. *t*-tests indicate *P* < 0.001.

The number of self progeny generated by WT hermaphrodites in this experiment (330 ± 40) was similar to the value of 327 reported by Hodgkin and Barnes (1991) and greater than the value of 263 reported by Hughes *et al.* (2007) and the value of 199 reported by Ward and Carrel (1979). The number of mated progeny observed in this experiment (710 ± 180) was lower than the value of 913 reported by Hodgkin and Barnes (1991) and greater than the value of 434 reported by Hughes *et al.* (2007) and the value of 479 reported by Ward and Carrel (1979). These differences in values might reflect genetic differences between WT strains due to spontaneous mutations that arise during laboratory cultivation, epigenetic differences between WT strains, or minor differences in culture conditions such as temperature, humidity, or nematode growth medium composition. These differences might also reflect variations in the approach to data analysis; for example, here we censored animals that displayed matricidal hatching, whereas animals that displayed matricidal hatching may have been included in some data sets.

To determine the effect of altering progeny production on somatic aging, we compared the spans of time self-fertile and mated WT hermaphrodites displayed coordinated body movement, pharyngeal pumping, and survival (see *Materials and Methods*) (Huang *et al.* 2004). For animals that died as a result of aging, the mean body movement span was decreased 8% in mated animals: 7.1 ± 2.1 d for mated animals and 7.7 ± 2.2 d for self-fertile animals (p_ANOVA_ = 0.16, p_log ANOVA_ = 0.14, p_Wilcoxon_ = 0.12, and p_log-rank_ = 0.20) (Figure 1E and Table 1B). The mean pharyngeal pumping span was increased 3% in mated animals: 11.9 ± 2.4 d for mated animals and 11.6 ± 3.8 d for self-fertile animals (p_ANOVA_ = 0.62, p_log ANOVA_ = 0.28, p_Wilcoxon_ = 0.45, p_log-rank_ = 0.35) (Figure 1G and Table 1B). The mean life span was decreased 9% in mated animals: 15.6 ± 3.3 d for mated animals and 17.1 ± 5.8 d for self-fertile animals (p_ANOVA_ = 0.08, p_log ANOVA_ = 0.23, p_Wilcoxon_ = 0.40, and p_log-rank_ = 0.014) (Figure 1I and Table 1B). The maximum life span was not analyzed because the sample size did not support a rigorous statistical analysis of this trait. The differences between mated and self-fertile animals in pharyngeal pumping span and body movement span were not significant based on all four statistical tests. The life span difference was not significant based on three statistical tests, although the log-rank test indicated the difference is significant using *P* < 0.05 as the standard. We interpret this life span difference as a trend that was not clearly statistically significant. The size of these samples, 64 mated hermaphrodites and 67 self-fertile hermaphrodites, provided at least 80% statistical power to detect a 15% mean difference in body movement span (±1.08 d), pharyngeal pumping span (±1.60 d), and life span (±2.37 d). This power analysis indicates these data sets are large enough to detect relatively small differences in somatic aging spans.

**Figure 2:**
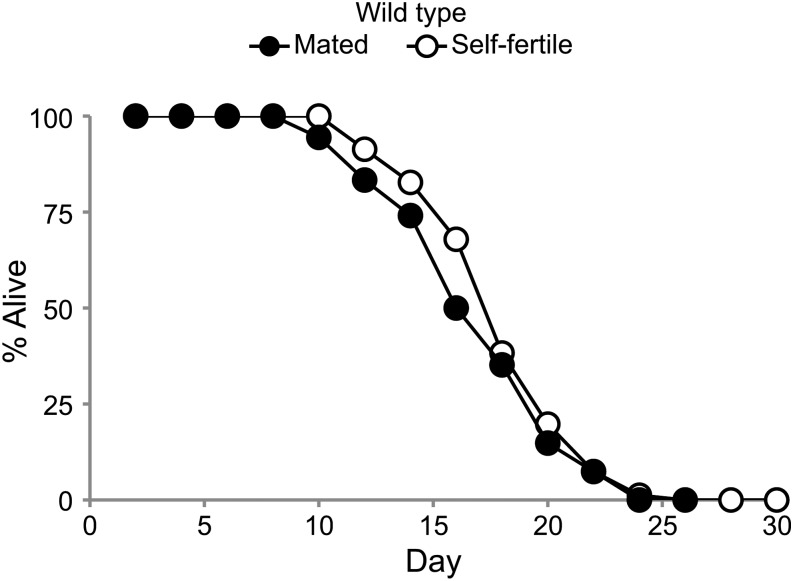
Self-fertile and mated WT hermaphrodites displayed similar life spans. Survival curves for WT hermaphrodites that were self fertile or mated to WT males for 24 hr on day 1 of adulthood and analyzed using a cross sectional study design.

To further analyze the life span of mated and self-fertile hermaphrodites, we conducted a cross sectional study. Self-fertile and mated hermaphrodites were analyzed by transferring animals to fresh dishes every two days until progeny production ceased and then monitoring survival. WT hermaphrodites were mated to WT males for 24 hr, and animals that generated male cross progeny the next day and did not subsequently die as a result of matricidal hatching were evaluated for life span. The mean life span of mated hermaphrodites (15.2 ± 3.8 d) was 6% shorter than the mean life span of self-fertile hermaphrodites (16.2 ± 3.3 d) (Figure 2 and Table 1A). The mean life spans of self-fertile and mated hermaphrodites were not significantly different using *P* < 0.05 as the standard (p_ANOVA_ = 0.11, p_log ANOVA_ = 0.08, p_Wilcoxon_ = 0.14, p_log-rank_ = 0.24). Similar to the results of the longitudinal study, in this cross sectional study the mated hermaphrodites displayed a life span that was slightly shorter than self-fertile hermaphrodites, but in this trial the difference was not significant according to any of the statistical tests. We interpret this life span difference as a trend that was not clearly statistically significant.

Mated and self-fertile WT hermaphrodites generated similar numbers of progeny for the first 2 d of adulthood; by contrast, mated WT hermaphrodites generated significantly more progeny than self-fertile hermaphrodites on days 3−10 (Figure 1A). These data suggest that substantially increasing the level of progeny production late in the reproductive period does not substantially alter somatic aging in WT hermaphrodites.

### Increased progeny production caused by mating self-sterile *fog-2* hermaphrodites did not accelerate somatic aging

To analyze hermaphrodites that do not display progeny production early or late in the reproductive period, we compared unmated *fog-2(q71)* females with *fog-2(q71)* females mated to WT males. Unmated *fog-2(q71)* females generate no self-sperm, lay very few unfertilized oocytes since the ovulation signal from sperm is missing, and produce no self progeny (McCarter *et al.* 1999). Mated *fog-2(q71)* females generate a similar number of cross progeny as mated WT hermaphrodites (Clifford *et al.* 2000) and may have altered physiology as a result of germline activity. As described for WT animals, we excluded animals that died because of internal hatching of progeny or accidental causes; we only analyzed mated animals that survived past the end of the reproductive period as documented by at least one day of postreproductive life span. Unmated *fog-2(q71)* females generated no progeny, whereas *fog-2(q71)* females mated to WT males generated 630 ± 180 progeny (Figure 1B and Table 1C). Unmated *fog-2(q71)* females displayed a reproductive span of 0 d, whereas *fog-2(q71)* females mated to WT males displayed a mean reproductive span of 7.7 ± 2.3 d (Figure 1D and Table 1C). Because unmated *fog-2(q71)* animals do not generate any progeny, a one-sample *t*-test, not ANOVA or Wilcoxon, is appropriate for determining statistical significance. Mated *fog-2(q71)* females generated significantly more progeny (p*_t_*_-test_ < 0.001) and reproduced for a significantly longer period of time (p*_t_*_-test_ < 0.001) than unmated *fog-2(q71)* females (Table 1C).

To determine whether progeny production affected somatic aging in *fog-2(q71)* females, we compared the body movement, pharyngeal pumping, and life spans of mated and unmated *fog-2(q71)* animals. The mean body movement span was increased 7% in mated animals: 5.8 ± 1.9 d for mated animals and 5.4 ± 1.7 d for unmated animals. The difference was not significant based on all four tests (p_ANOVA_ = 0.31, p_log ANOVA_ = 0.36, p_Wilcoxon_ = 0.34, and p_log-rank_ = 0.27; Figure 1F and Table 1C). The mean pharyngeal pumping span was increased 11% in mated animals: 11.3 ± 3.3 d for mated animals and 10.2 ± 3.9 d for unmated animals. The difference was not significant on the basis of all four tests (p_ANOVA_ = 0.15, p_log ANOVA_ = 0.09, p_Wilcoxon_ = 0.07, and p_log-rank_ = 0.46) (Figure 1H and Table 1C). The mean life span was increased 13% in mated animals: 16.3 ± 4.6 d for mated animals and 14.4 ± 4.5 d for unmated animals. The difference was significant based on two statistical tests (p_log ANOVA_ = 0.04, and p_Wilcoxon_ = 0.04) and was not significant based on two statistical tests (p_ANOVA_ = 0.07 p_log-rank_ = 0.18) (Figure 1J and Table 1C). Thus, there was a trend toward a longer life span in mated animals, but this difference was not clearly statistically significant. The size of these samples, 45 mated animals and 41 unmated animals, provided at least 80% statistical power to detect a 20% mean difference in body movement span (±1.12 days), pharyngeal pumping span (±2.23 d), and life span (±2.82 d). This power analysis indicates our data sets are large enough to detect relatively small differences in somatic aging spans. Together, these data indicate that the physiological differences caused by male mating, including significantly increased levels of early and late progeny production, did not accelerate somatic aging in *fog-2(q71)* hermaphrodites.

In addition to allowing a comparison of genetically identical animals that generated different numbers of progeny as a result of male mating, these data can be used to compare unmated *fog-2(q71)* animals that generated no progeny with self-fertile WT animals that generated 330 ± 40 self progeny early in the reproductive period. This comparison does not involve the variable of male mating. Self-fertile WT animals displayed small but statistically significant extensions of mean body movement span (p_ANOVA_ < 0.001, p_log ANOVA_ < 0.001, and p_Wilcoxon_ < 0.001), pharyngeal pumping span (p_ANOVA_ = 0.06, p_log ANOVA_ = 0.03, and p_Wilcoxon_ = 0.03), and life span (p_ANOVA_ = 0.02, p_log ANOVA_ = 0.01, and p_Wilcoxon_ = 0.02) (Figure 1, E−J and Table 1). These data indicate that high levels of progeny production early in the reproductive period do not accelerate somatic aging and may even delay somatic aging; however, differences between WT animals and *fog-2(q71)* animals could reflect pleiotropic effects of the *fog-2* mutation in addition to reduced progeny production.

### Progeny production and somatic aging were positively correlated in mated WT hermaphrodites

WT animals were analyzed using a longitudinal study design, a method characterized by repeated measurements of a specific trait on the same individual (Miller 1997; Huang *et al.* 2004). Longitudinal studies take advantage of variation among individuals, and it is relevant to consider the underlying basis for this variation. Because WT *C. elegans* are genetically identical, the differences cannot be attributed to genetic variation. One possible source of variation is stochastic differences during development and adult life, which have been suggested to cause individual variation in aging systems (Sozou and Kirkwood 2001; Herndon *et al.* 2002; Kirkwood *et al.* 2005; Shoyama *et al.* 2007). A second possible source is variable environmental exposures; although all the animals were cultured on agar dishes with a lawn of *E. coli*, these dishes do not have a uniform surface, and animals may spend more or less time in slightly different environments. Each individual was monitored daily for progeny production, coordinated body movement, pharyngeal pumping, and survival. Since these measurements were made non-invasively, we were able to collect a complete data set for each individual and exclude individuals that died prematurely from causes not related to aging. To analyze these data, we used the Pearson correlation analysis after log transformation of the data to approximate a normal data distribution. A score of +1 indicates a perfect positive correlation, a score of -1 indicates a perfect negative correlation, and a score of 0 indicates no correlation (Kendall and Gibbons 1990). A positive correlation indicates that the two traits share a common regulatory mechanism or are connected by a causal pathway.

**Figure 3:**
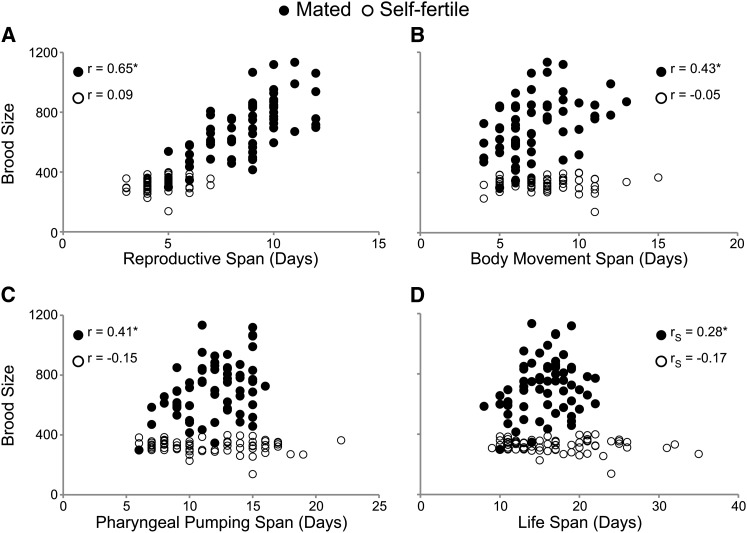
Increased brood size was correlated with delayed somatic aging in mated hermaphrodites. Scatter plots comparing brood size with reproductive and somatic aging in mated (closed circle) and self-fertile (open circle) WT hermaphrodites. Each data point represents the brood size and (A) reproductive span, (B) body movement span, (C) pharyngeal pumping span, and (D) life span for an individual animal. Pearson correlation coefficients are denoted by r values. *P < 0.05. Number of animals: WT self-fertile = 67, WT mated = 64.

If progeny production causes an acceleration of somatic aging, then longitudinal studies are predicted to reveal a negative correlation between progeny production and somatic aging. In contrast to this prediction, the brood size of mated WT hermaphrodites was positively and significantly correlated with reproductive (r = 0.65), body movement (r = 0.43), pharyngeal pumping (r = 0.41), and life (r = 0.28) spans (Figure 3, Table 2, and Table S1). Furthermore, the mated reproductive span was positively and significantly correlated with pharyngeal pumping span (r = 0.25) (Table 2 and Table S1). The mated reproductive span was also positively correlated with the body movement and life spans, although not statistically significant. These data indicate that mated animals with larger broods have longer reproductive periods. Furthermore, mated animals with larger broods move, pump, and live longer than those with smaller broods, indicating that total brood size in mated animals is predictive of somatic aging.

**Table 2:** Pearson correlation values for WT hermaphrodites

	RS	MS	PS	LS
Self-fertile*^a^*				
Brood	**0.09 (0.455)**	−**0.05 (0.670)**	−**0.15 (0.229)**	−**0.17 (0.174)**
RS	−	−0.08 (0.530)	−0.06 (0.635)	−0.03 (0.837)
MS	−	−	0.55 (<0.001)	0.44 (<0.001)
PS	−	−	−	0.86 (<0.001)
Mated to a WT*^a^*				
Brood	**0.65 (<0.001)**	**0.43 (<0.001)**	**0.41 (<0.001)**	**0.28 (0.023)**
RS	−	0.15 (0.226)	0.25 (0.049)	0.11 (0.403)
MS	−	−	0.40 (<0.001)	0.21 (0.088)
PS	−	−	−	0.87 (<0.001)
Mated to a *lov-1(sy582)**^a^*				
Brood	0.15 (0.585)	0.30 (0.275)	−0.14 (0.625)	0.02 (0.930)
RS	−	0.22 (0.438)	0.48 (0.068)	0.50 (0.060)
MS	−	−	0.44 (0.105)	0.70 (0.004)
PS	−	−	−	0.88 (<0.001)
Mated to a *fer-6(hc6)**^a^*				
Brood	0.36 (0.220)	−0.17 (0.580)	0.04 (0.900)	0.12 (0.697)
RS	−	−0.38 (0.198)	−0.21 (0.487)	−0.30 (0.325)
MS	−	−	0.54 (0.057)	0.52 (0.071)
PS	−	−	−	0.90 (<0.001)

Each line shows the Pearson correlation value (r) between the two indicated life-history traits. *P* values are shown in parentheses. Values in bold indicate the data depicted in Figure 3. WT, wild type; RS, reproductive span; MS, body movement span; PS, pharyngeal pumping span; LS, life span; brood, total number of live progeny.

a WT hermaphrodites were self-fertile (unmated) or mated to WT, *lov-1(sy582)*, or *fer-6(hc6)* males.

The brood size of self-fertile WT animals was not significantly correlated with reproductive span, body movement span, pharyngeal pumping span or life span (Figure 3, Table 2, and Table S1). Consistent with our previous results, the self-fertile reproductive span was not significantly correlated with body movement, pharyngeal pumping, or life spans (Table 2 and Table S1) (Huang *et al.* 2004). Self-fertile hermaphrodites generate small broods as a result of sperm depletion, and they are an important control demonstrating that positive correlations are not inherent in our study design.

In self-fertile animals, the body movement, pharyngeal pumping, and life spans were positively and significantly correlated with one another (Table 2 and Table S1). Mated hermaphrodites displayed a similar pattern, although the positive correlation between body movement and life span was not statistically significant. These results indicate that age-related declines in body movement, pharyngeal pumping, and survival may share a common mechanism (Huang *et al.* 2004).

Compared with self-fertile animals, mated hermaphrodites were exposed to WT males for one day to acquire male sperm in order to increase progeny production. To test the possibility that exposure to males or the act of copulation has an effect independent of increased progeny production, we conducted longitudinal studies of WT hermaphrodites mated to *lov-1(sy582)* or *fer-6(hc6)* males. *lov-1(sy582)* males initiate the mating ritual but rarely transfer sperm (Barr and Sternberg 1999), whereas *fer-6(hc6)* males copulate with the hermaphrodite and transfer seminal fluid, but do not transfer functional sperm (Ward *et al.* 1981; Gems and Riddle 1996). The brood size, pharyngeal pumping span, and life span of WT hermaphrodites mated to *lov-1(sy582)* or *fer-6(hc6)* males were not significantly different from self-fertile hermaphrodites, whereas the reproductive span was slightly reduced (Table 3). A Pearson correlation analysis showed that progeny production in hermaphrodites mated to *lov-1(sy582)* or *fer-6(hc6)* males was not significantly correlated with body movement, pharyngeal pumping, or life spans (Table 2). Therefore, contact with males, the act of copulation, and transfer of seminal fluid were not sufficient to substantially change the pattern of correlations between brood size and somatic aging observed in self-fertile hermaphrodites.

**Table 3:** Summary statistics of WT hermaphrodites mated to mutant males

	Brood	RS	MS	PS	LS	N*^a^*
Self-fertile	330 ± 40	4.6 ± 0.9	7.7 ± 2.2	11.6 ± 3.8	17.1 ± 5.8	67
* lov-1**^b^*	340 ± 30	3.6 ± 0.5*	6.0 ± 1.1*	12.2 ± 4.4	17.2 ± 7.0	13
* fer-6**^b^*	320 ± 30	3.2 ± 0.4*	6.7 ± 2.5	12.4 ± 3.6	17.8 ± 6.5	15

Values represent the mean ± SD. Brood size is measured in number of progeny. Spans are measured in days. The self-fertile data are the same as in Table 1. WT, wild type; brood, total number of live progeny; RS, reproductive span; MS, body movement span; PS, pharyngeal pumping span; LS, life span; ANOVA, analysis of variance.

a Number of animals analyzed.

b WT hermaphrodites were self-fertile or mated to *lov-1(sy582)* or *fer-6(hc6)* males.

* *P* < 0.05 compared with self-fertile hermaphrodites by ANOVA.

### Progeny production on specific days was positively correlated with life span

**Figure 4:**
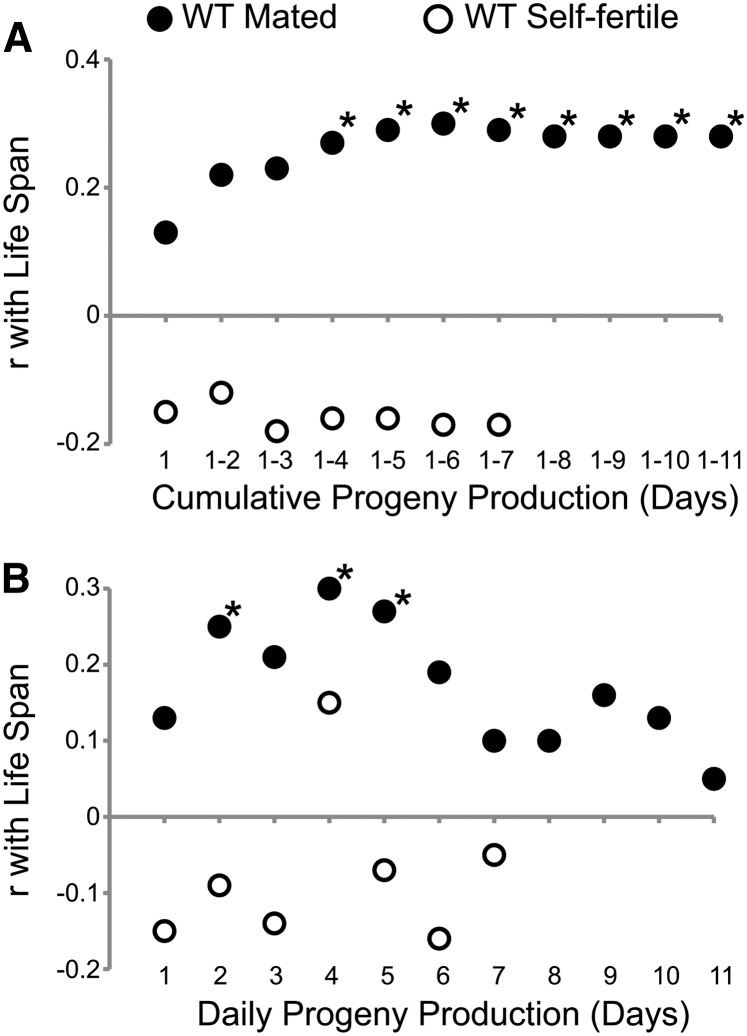
Correlations between progeny production and life span in mated and self-fertile WT animals. Pearson rank correlation values (r) were plotted *vs.* (A) cumulative progeny production or (B) single-day progeny production. *P < 0.05. Number of animals: WT self-fertile = 67, WT mated = 64.

Cumulative progeny production from day 1 to 11 was positively correlated with delayed somatic aging in mated hermaphrodites. To determine the earliest time that cumulative progeny production beginning on day 1 is correlated with delayed somatic aging, we conducted Pearson correlation analyses after log transformation of the data to approximate a normal data distribution. Cumulative progeny production of mated WT hermaphrodites was positively correlated with body movement, pharyngeal pumping and life spans for every day of the reproductive period (Table 4 and Table S2). The values were statistically significant for body movement span starting on day 1 (r = 0.41−0.52), for pharyngeal pumping span starting on day 2 (r = 0.34−0.41), and for life span starting on day 4 (r = 0.27−0.30) (Figure 4A, Table 4, and Table S2). Most of these positive correlations were also significant (*P* < 0.05) after the stringent Bonferroni correction for multiple comparisons was applied. These data indicate that cumulative progeny production after 4 d is a positive predictor of body movement, pharyngeal pumping, and life spans. In contrast, the cumulative progeny production of self-fertile animals was not significantly correlated with body movement, pharyngeal pumping, or life span on any day (Figure 4A; Table 4, and Table S2). These data indicate that the positive correlations observed in mated animals reflect a specific relationship between high levels of progeny production early in life and longevity.

**Table 4:** Pearson correlation values for cumulative progeny production

	Cumulative Progeny Production (Days)										
	1	1−2	1−3	1−4	1−5	1−6	1−7	1−8	1−9	1−10	1−11
Mated											
RS	0.13	0.17	0.30*	0.33*	0.42*	0.52**	0.59**	0.63**	0.64**	0.65**	0.65**
MS	0.41**	0.52**	0.50**	0.51**	0.48**	0.45**	0.44**	0.43**	0.43**	0.43**	0.43**
PS	0.21	0.34*	0.35*	0.38**	0.39**	0.40**	0.40**	0.41**	0.41**	0.41**	0.41**
LS	0.13	0.22	0.23	0.27*	0.29*	0.30*	0.29*	0.28*	0.28*	0.28*	0.28*
Self-fertile											
RS	−0.19	−0.27*	0.00	0.05	0.07	0.09	0.09	−	−	−	−
MS	−0.08	0.02	−0.04	−0.05	−0.05	−0.05	−0.05	−	−	−	−
PS	−0.11	−0.07	−0.16	−0.14	−0.14	−0.15	−0.15	−	−	−	−
LS	−0.15	−0.12	−0.18	−0.16	−0.16	−0.17	−0.17	−	−	−	−

Numbers are the Pearson correlation value between the indicated span and the cumulative progeny production during the indicated days. RS, reproductive span; MS, body movement span; PS, pharyngeal pumping span; LS, life span.

* *P* < 0.05.

** *P* < 0.05 after Bonferroni correction.

We determined whether progeny production on individual days was predictive of somatic aging. A Pearson correlation analysis demonstrated progeny production of mated animals on days 1, 2, 3, and 4 was significantly positively correlated with body movement span (r = 0.37−0.53); mated progeny production on days 2, 3, 4, 5, 6, 9, and 10 was significantly positively correlated with pharyngeal pumping span (r = 0.26−0.37); and mated progeny production on days 2, 4, and 5 was significantly positively correlated with life span (r = 0.25−0.30) (Figure 4B, Table 5, and Table S3). Six of these 14 positive correlations also were significant (*P* < 0.05) after the Bonferroni correction for multiple comparisons was applied. By contrast, daily progeny production of self-fertile animals was not significantly correlated with life span or other measures of somatic aging (Figure 4B, Table 5, and Table S3). These data indicate that the number of progeny generated by mated *C. elegans* hermaphrodites on several days is an early biomarker that is a positive predictor of delayed somatic aging.

**Table 5:** Pearson correlation values for daily progeny production

	Daily Progeny Production (Days)										
	1	2	3	4	5	6	7	8	9	10	11
Mated											
RS	0.13	0.15	0.41**	0.29*	0.54**	0.79**	0.90**	0.89**	0.80**	0.67**	0.53**
MS	0.41**	0.53**	0.37**	0.42**	0.24	0.17	0.14	0.13	0.24	0.23	0.24
PS	0.21	0.37**	0.29*	0.37**	0.31*	0.26*	0.22	0.23	0.28*	0.26*	0.20
LS	0.13	0.25*	0.21	0.30*	0.27*	0.19	0.10	0.10	0.16	0.13	0.05
Self-fertile											
RS	−0.19	−0.25*	0.30*	0.41*	0.68*	0.63*	0.43*	—	—	—	—
MS	−0.08	0.05	−0.09	−0.02	−0.07	0.00	0.06	—	—	—	—
PS	−0.11	−0.04	−0.16	0.15	−0.07	−0.18	0.01	—	—	—	—
LS	−0.15	−0.09	−0.14	0.15	−0.07	−0.16	−0.05	—	—	—	—

Numbers are the Pearson correlation value between the indicated span and the daily progeny production on the indicated days. RS, reproductive span; MS, body movement span; PS, pharyngeal pumping span; LS, life span.

* *P* < 0.05.

** *P* < 0.05 after Bonferroni correction.

One interpretation of the positive correlations between progeny production and somatic aging spans is that mated animals that live longer have more time to generate progeny. For this interpretation to be valid, some animals must die during the reproductive period. To exclude this interpretation, we only analyzed mated animals that survived beyond the reproductive period. Furthermore, we observed that cumulative progeny production on days 1−4 was positively correlated with the body movement, pharyngeal pumping, and life spans, whereas the first animal in this group died on day 8. Thus, these positive correlations cannot be attributed to longer-lived animals having more time to generate progeny.

### Early progeny production was positively correlated with late progeny production

**Figure 5:**
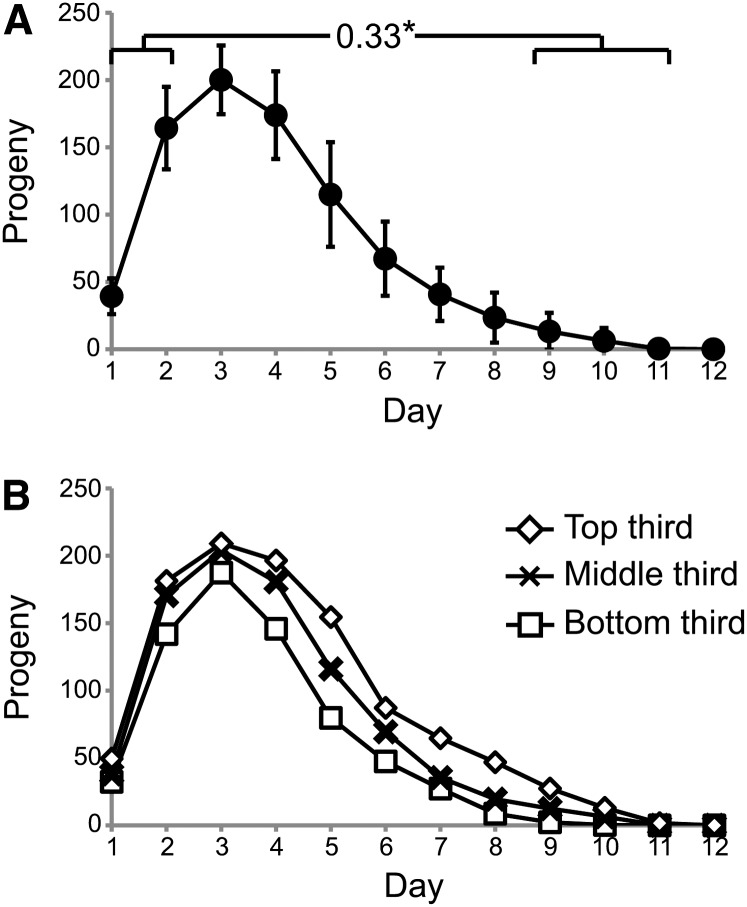
Correlations between early and late reproduction in mated WT animals. (A) Daily progeny production of mated WT hermaphrodites. Brackets indicate early reproduction (days 1–2) and late reproduction (days 9–11) displayed a positive Pearson rank correlation value. *P < 0.05. (B) A population of mated hermaphrodites was analyzed by dividing them into three groups on the basis of the total brood size. The graphs show average daily progeny production of low-, middle-, and high-reproducing groups. Number of animals = 64

To test the relationship between early and late reproduction in individuals, we used Pearson correlations to analyze these characteristics. Mated animals produce ~25% of total progeny on days 1−2 and <5% on days 9−11. Comparing cumulative progeny production on days 1−2 with days 9−11 by Pearson correlation analysis revealed a significant positive correlation (r = 0.33) (Figure 5A, Table 6, and Table S4). To determine how the selection of specific days for early and late reproduction affected this relationship, we analyzed early reproduction defined as cumulative progeny production on days 1−2, 1−3, 1−4, or 1−5 with late reproduction defined as cumulative progeny production on days 6−11, 7−11, 8−11, 9−11, or 10−11. All 20 Pearson correlation values were positive; 17 values were significant based on the standard statistical test (r = 0.30−0.58), and 15 of these values were statistically significant after applying the Bonferroni correction for multiple comparisons (Table 6 and Table S4). These results indicate that there is a robust positive correlation between early and late reproduction that is independent of the precise definition of early and late.

**Table 6:** Pearson correlation values comparing early and late reproduction

	Cumulative Late Reproduction (Days)*^a^*				
	6−11	7−11	8−11	9−11	10−11
Cumulative Early Reproduction (Days)*^b^*					
1−2	0.21	0.24	0.23	0.33**	0.30*
1−3	0.38**	0.36**	0.33**	0.38**	0.33**
1−4	0.45**	0.40**	0.35**	0.38**	0.31*
1−5	0.58**	0.50**	0.44**	0.43**	0.33**

Numbers are the Pearson correlation values (r).

a Cumulative progeny production for each animal was calculated from the indicated start point through day 11.

b Cumulative progeny production for each animal was calculated from day 1 through the indicated endpoint.

* *P* < 0.05.

** *P* < 0.05 after Bonferroni correction.

To further evaluate the relationships between early and late reproduction, we used an independent approach to analyze these data. Animals were divided into three groups—top, middle, and lower thirds—on the basis of total progeny production. The group with the greatest levels of early reproduction also displayed the highest levels of late reproduction, whereas the group with the lowest level of early progeny production also displayed the lowest levels of late reproduction (Figure 5B). These data demonstrate that early and late progeny production are positively correlated and indicate that lower levels of early progeny production do not promote high levels of late reproduction.

## Discussion

### High levels of progeny production were positively correlated with extended reproductive span and life span in mated hermaphrodites

Longitudinal studies are a powerful approach to define the relationships between age-related changes and other life-history traits. We previously used longitudinal studies to demonstrate that multiple somatic aging traits, including coordinated body movement span, pharyngeal pumping span, and life span, are positively correlated in self-fertile *C. elegans* hermaphrodites (Huang *et al.* 2004). We also demonstrated that the self-fertile reproductive span was not an accurate measure of reproductive aging because self-fertile animals cease progeny production as a result of sperm depletion (Hughes *et al.* 2007). By contrast, mated reproductive span is an indication of reproductive aging, because mated hermaphrodites are not sperm limited and cease progeny production as a result of age-related degeneration of the reproductive system (Ward and Carrel 1979; Hughes *et al.* 2007). Here we used longitudinal studies to define relationships between progeny production, reproductive aging, and somatic aging in mated WT *C. elegans* hermaphrodites that were not sperm limited. All of the animals that we analyzed survived beyond the time of reproductive cessation, so all of these animals displayed a complete age-related decline of progeny production. If progeny production causes age-related degeneration of the reproductive system, then animals that generate more progeny are predicted to have accelerated reproductive aging and a shorter reproductive span. However, our data revealed a positive correlation between progeny production and reproductive span−mated animals that generated the most cross progeny also reproduced for the longest period. This positive correlation indicates that either high levels of progeny production cause extended reproduction or a common cause promotes both processes.

Total progeny production of mated hermaphrodites was also positively correlated with body movement span, pharyngeal pumping span, and life span. Thus, mated animals that generated more cross progeny displayed longer periods of body movement, pharyngeal pumping, and survival. These findings indicate that progeny production does not cause somatic aging; to the contrary, either high levels of cross-progeny production extend health span or a common mechanism mediates progeny production and health span. Consistent with these results, our data revealed that reproductive aging, as measured by reproductive span, was positively correlated with somatic aging, as measured by pharyngeal pumping span. Overall, the results indicate that there is a positive relationship between high levels of progeny production, delayed reproductive aging, and delayed somatic aging—mated animals that generated more cross progeny reproduced and lived longer than mated animals that generated fewer cross progeny.

Relationships between progeny production and somatic aging have been analyzed in a variety of conditions. Studies examining progeny production in self-fertile hermaphrodites (Klass 1977; Chen *et al.* 2007) and briefly mated hermaphrodites (Gems and Riddle 1996) indicated life span was not correlated with brood size. Consistent with these findings, we observed no correlation between brood size and life span in self-fertile hermaphrodites. Relationships between reproductive aging and somatic aging have also been analyzed in self-fertile hermaphrodites. In self-fertile, WT hermaphrodites (Huang *et al.* 2004) or recombinant inbred *C. elegans* lines (Johnson 1987) the self-fertile reproductive span was not correlated with various measures of somatic aging, including life span. Consistent with these studies, we observed that the reproductive span was not consistently correlated with the life span of self-fertile hermaphrodites.

In contrast to self-fertile animals, mated hermaphrodites displayed positive correlations between brood size and life span and between reproductive span and life span. What accounts for this difference between self-fertile and mated hermaphrodites? The likely reason is that brood size and reproductive span in self-fertile animals are a reflection of sperm depletion, whereas brood size and reproductive span in mated hermaphrodites are a reflection of reproductive aging. Thus, studies of self-fertile animals do not detect underlying relationships between reproduction and aging that are apparent in mated hermaphrodites.

### A common mechanism regulates reproduction and longevity

Two basic models can explain the positive correlation between high levels of progeny production and delayed somatic aging: (1) progeny production causes health-span extension or (2) a common cause mediates high levels of progeny production and health span extension. For example, an endocrine factor might be a common mediator of progeny production and life span. To distinguish between these models, we manipulated levels of progeny production by controlling sperm availability. For WT animals, self-fertile hermaphrodites that generated about 330 progeny and mated hermaphrodites that generated ~710 progeny displayed similar age-related declines in somatic function. For *fog-2(q71)* animals that do not self fertilize, unmated females that generated no progeny and mated females that generated ~630 progeny displayed similar age-related declines in somatic function. If progeny production caused life-span extension, then mated animals should live longer than unmated animals. Our results are not consistent with this prediction, suggesting that a common cause mediates reproduction and longevity. Our data do not identify the common cause, but it cannot be a genetic difference because the longitudinal studies were conducted with genetically identical individuals. Thus the common cause may be related to environmental differences experienced by individuals or stochastic events that occur during development and/or adulthood that ultimately affect viability (Sozou and Kirkwood 2001; Herndon *et al.* 2002; Kirkwood 2005; Shoyama *et al.* 2007). These results provide a foundation for future studies to elucidate the common cause linking reproduction and longevity.

### Progeny production does not accelerate somatic aging in mated *C. elegans* hermaphrodites

Because some theories of aging postulate that reproduction causes age-related degeneration, an important issue in aging research is determining experimentally how reproduction affects reproductive and somatic aging. Several approaches have been used to address this issue in *C. elegans*, an important model organism for studies of aging. One approach is to compare sterile mutant animals to fertile, WT animals. If progeny production accelerates somatic aging, then sterile mutant animals are predicted to display an extended life span. However, sterile mutant animals that are defective in sperm production (Klass 1977; Kenyon *et al.* 1993; Arantes-Oliveira *et al.* 2002), or in which oocyte precursor cells undergo apoptosis (Arantes-Oliveira *et al.* 2002) display similar life spans to fertile WT animals. Here we showed that *fog-2(q71)* sterile animals did not display an extended life span compared with self-fertile WT animals, consistent with a previous report (Arantes-Oliveira *et al.* 2002). These results suggest that progeny production may not accelerate *C. elegans* aging. However, a caveat to this interpretation is that these mutations may have effects in addition to sterility, and these pleiotropic effects might abrogate a life span extension. In contrast, sterile animals that are defective in germline stem cell proliferation display an extended life span compared to WT animals (Arantes-Oliveira *et al.* 2002), as do *spe-26* mutant animals that are defective in sperm production (Van Voorhies 1992). These results suggest that progeny production may accelerate aging. This interpretation is supported by the numerous findings that mutations that extend life span also reduce progeny production (Chen *et al.* 2007; Kenyon 2010). However, a caveat to this interpretation is that the mutations may reduce fertility but cause independent pleiotropic effects that promote life-span extension.

To address these interpretation issues, Kenyon and colleagues induced sterility by laser ablation of the germline stem cells and/or the somatic gonad in genetically identical animals (Kenyon *et al.* 1993; Hsin and Kenyon 1999; Arantes-Oliveira *et al.* 2002). These studies indicated that sterile, gonad-ablated animals have a WT life span, suggesting that progeny production does not accelerate aging. Furthermore, ablation of the germline stem cells in animals with an intact somatic gonad sterilizes animals and extends life span. These data led to the model that the germline stem cells are the source of a signal that accelerates aging, whereas the somatic gonad is the source of a signal that delays aging (Hsin and Kenyon 1999). Furthermore, Gems and Riddle (1996) analyzed hermaphrodite life span when co-cultured with males and concluded that the physical stress of mating, not increased gamete production, reduced hermaphrodite life span. These data led to the conclusion that there is no apparent trade-off between longevity and increased egg production.

Here, we show that increasing progeny production by manipulating sperm availability did not substantially alter life span. First, self-fertile WT hermaphrodites that generated progeny early in the reproductive period but no progeny late in the reproductive period were compared with mated, WT hermaphrodites that generated progeny early and late in the reproductive period. Although the mated hermaphrodites generated an average of 380 more progeny than self-fertile hermaphrodites, the mean body movement span, pharyngeal pumping span, and life span were similar. Second, sterile, unmated *fog-2(q71)* females that generated no progeny were compared with mated *fog-2(q71)* females that generated progeny early and late in the reproductive period. Although the mated animals generated an average of 630 progeny, the mean body movement span, pharyngeal pumping span and life span were similar to sterile animals. By comparing genetically identical animals, in these experiments we overcome the limitation of interpreting differences between sterile mutant animals and WT control animals. We also compared sterile, unmated *fog-2(q71)* females that generated no progeny with self-fertile WT hermaphrodites that produced progeny early in the reproductive period but no progeny late in the reproductive period. Although WT animals generated an average of 330 progeny, this strain did not display a shortened life span compared with *fog-2* animals that generated no progeny. These results indicate that progeny production does not accelerate or retard age-related changes that control longevity. These findings are consistent with the longitudinal studies showing that progeny production is positively correlated with longevity.

To focus on animals that completed reproduction and died as a result of age-related degeneration, we excluded animals that died during the reproductive period as a result of matricidal hatching. Matricidal hatching occurs when egg laying is delayed and larvae hatch internally, leading to the death of the hermaphrodite. Matricidal hatching is infrequent in self-fertile hermaphrodites but frequently affects mated hermaphrodites (Pickett and Kornfeld 2013), and thus a substantial number of mated hermaphrodites were excluded based on this criterion. Therefore, the conclusions of this study apply to the subset of the population that escaped matricidal hatching.

### Progeny production is an early-life predictor of delayed somatic aging

The identification of biomarkers of aging is an important goal of aging research because biomarkers can facilitate experimental work, help elucidate mechanisms of age-related degeneration, and are useful for preventative treatment strategies. Several biomarkers of *C. elegans* hermaphrodite aging have been described, including the extent of muscle degeneration, increased cuticle and yolk generation (Herndon *et al.* 2002), the accumulation of age pigments (Gerstbrein *et al.* 2005), expression patterns of specific microRNAs (Pincus *et al.* 2011), and the level of induction of P*hsp-16.2*::*GFP* in response to heat shock on day 1 of adulthood (Rea *et al.* 2005). We previously used longitudinal studies, a rigorous method to determine predictive measures of life span, to demonstrate that fast body movement span (mean 8.2 ± 1.7 d), fast pharyngeal pumping span (mean 8.1 ± 2.1 d), and pharyngeal pumping span (mean 11.3 ± 3.0 d) are predictive of life span in self-fertile WT hermaphrodites (Huang *et al.* 2004). In the current study, we demonstrated that cumulative progeny production, which can be scored on day 11, was predictive of somatic aging in mated hermaphrodites. Furthermore, cumulative progeny production through day 4 and progeny production on days 2, 4, and 5 alone were predictive of life span. These results are a significant advance in two ways. First, reproductive function has not been previously reported to be predictive of life span in *C. elegans*, and we are not aware of this relationship being described for other animals. Thus, the longitudinal study approach has revealed a novel understanding of a function that is related to longevity. Second, progeny production prior to day 5 is an early life event that can be scored before the body movement and pharyngeal pumping spans.

The utility of biomarkers is affected by several factors including ease of scoring, stage of scoring during the life span, and robustness of correlation with life span. Limitations of using cross progeny production as a biomarker include the need to mate hermaphrodites to males, which is a limitation because aging studies in *C. elegans* frequently are conducted with self-fertile animals, and the need to count progeny for one or more days. Nevertheless, this biomarker may be useful under specific circumstances.

### Early progeny production did not accelerate reproductive aging in mated *C. elegans* hermaphrodites

The relationships between early and late reproduction are important to understand but have not been well defined experimentally. Extensive use of the reproductive system early in life could (1) be an indication of the robustness of the system and/or (2) damage the system by use-related mechanisms or resource depletion, thereby limiting late reproduction. We previously addressed these relationships by using sperm availability to manipulate the level of early reproduction. We showed that for WT, *spe-8*, and *fog-2* animals, the level of early reproduction did not substantially affect the level of late reproduction when we compared animals with identical genotypes, leading to the conclusion that early progeny production neither accelerated nor delayed reproductive aging (Hughes *et al.* 2007). Here, using a longitudinal study, we observed that high levels of early progeny production were positively correlated with high levels of late progeny production. These data support the model that early progeny production does not cause a reduction of late progeny production, consistent with the conclusions of our previous work (Hughes *et al.* 2007). Mendenhall *et al.* (2011) observed that WT animals that were self-fertile early in life and mated late in life generated fewer late cross progeny than *fog-2(q71)* females that were sterile early in life and mated late in life, leading to the conclusion that self-progeny production early in life reduces the ability of animals to generate cross-progeny late in life. The longitudinal analysis of genetically identical animals described here is a different approach than comparing genetically identical animals that differ by sperm availability (Hughes *et al.* 2007) or comparing two different genotypes (Mendenhall *et al.* 2011). Thus, these results make a unique contribution to the experimental data addressing the connection between early and late reproduction.

The positive correlation between high levels of early and late reproduction indicates that these two processes are linked in a causal sequence or share a common cause. Although our data do not establish the basis for this correlation, it is instructive to consider one example of a common cause model: greater levels of male sperm may be a common cause that promotes higher levels of early and late progeny production. Although this study did not involve a measurement of male sperm levels in mated hermaphrodites, the study design did ensure that every mated hermaphrodite had an excess of male sperm, since hermaphrodites that reverted to using self sperm were excluded from the data analysis. Differences in the level of early progeny production are the result of differences in the rate of egg laying—high-producing hermaphrodites lay eggs at a faster rate than low-producing hermaphrodites. Ward and Carrel (1979) reported that mated hermaphrodites and self-fertile hermaphrodites lay eggs at the same rate early in the reproductive period. Thus, mating hermaphrodites to males, which increases sperm number, does not affect the rate of egg-laying. These findings indicate that differences between individual hermaphrodites in the amount of excess male sperm may not be the common cause for the observed correlation. Further studies are necessary to identify the basis for this positive correlation.

### Reproductive aging is an important component of evolutionary theories of aging

Progeny production and reproductive aging are important components of evolutionary theories of aging, and it is useful to place our study in the context of these theories. However, because our studies focus on mechanistic and physiological factors, rather than genetic changes, the results do not lead to definitive conclusions regarding evolutionary theories of aging.

Trade-off theories are based on the assumption that reproductive aging is a deleterious trait that reduces individual fitness by reducing progeny production. The antagonistic pleiotropy theory predicts that mutations promoting early reproduction confer a selective advantage even if they have late-acting deleterious effects such as reducing late reproduction or causing reproductive and somatic aging (Williams 1957). The disposable soma theory postulates that individuals invest metabolic resources into early reproduction at the expense of the long-term health of reproductive and somatic tissues, which need the same resources to prevent damage accumulation and age-related degeneration (Kirkwood 1977). The early-acting beneficial mutations proposed by these theories are maintained in populations despite their subsequent deleterious effects due to the diminishing effectiveness of natural selection as reproductive individuals age (Hamilton 1966; Charlesworth 1980). Experimental evidence interpreted as supporting trade-off theories include (1) single-gene mutations that reduce early progeny production and extend longevity (Walker *et al.* 2000; Kenyon 2010) and (2) selection experiments for enhanced late reproduction in *Drosophila* that resulted in lower levels of early reproduction (Rose and Charlesworth 1981b; Sgro and Partridge 1999).

Multilevel selection theories propose that individual reproductive restraint is an adaptive trait that regulates population density. Reproductive restraint maximizes population stability through optimization of individuals’ reproductive output to prevent overexploitation of the environment (Wynne-Edwards 1962; Wade 1980). Modeling studies propose that reproductive restraint may arise through communication comprised of a signal indicating overcrowding and an evolved response to this signal (Werfel and Bar-Yam 2004). *C. elegans* communicate overcrowding through the release of dauer pheromone (Cassada and Russell 1975; Golden and Riddle 1982, 1984), and the dauer larvae stage is nonreproductive, indicating that aspects of this model may be relevant to *C. elegans*. A criticism of multilevel selection theories is that unrestrained individuals will generate more progeny and have a selective advantage over restrained individuals (Kirkwood 2005). However, modeling studies suggest the emergence of unrestrained individuals is generally not a threat to populations exhibiting reproductive restraint (Wilson 1975; Werfel and Bar-Yam 2004). We proposed the optimal progeny number hypothesis that postulates reproductive aging is a mechanism of reproductive restraint, and thus an adaptive trait (Hughes *et al.* 2007). This hypothesis suggests the rate of reproductive aging is selected during evolution to achieve the optimal number of progeny for a specific environmental niche. For example, in an environmental niche where the optimal progeny number is high, selection will favor animals with delayed reproductive aging.

Conversely, selection will favor animals with accelerated reproductive aging if the optimal progeny number is low. In other words, selection will favor animals that generate the optimal number of progeny because this trait will maximize the number of animals in the population without overexploitation of the environmental niche. The positive correlations between reproductive and somatic aging as well as progeny production and somatic aging reported here are consistent with reproductive restraint and the optimal progeny number hypothesis. These results are also consistent with studies of reproduction and life span in other species, such as ants (Schrempf *et al.* 2005), fish (Reznick *et al.* 2004), and birds (McCleery *et al.* 2008). Together, these data indicate that species from a variety of taxa share the property that individuals can increase progeny production while delaying reproductive and somatic aging.

In the present study, we demonstrate that progeny production and life span in mated, WT *C. elegans* hermaphrodites are positively correlated under standard lab conditions. These results are consistent with the optimal progeny number hypothesis. However, because traits sculpted by evolution have a genetic basis, evaluating the existence of evolutionarily derived trade-offs requires an evaluation of the underlying genetic architecture (Reznick 1985). Our results are based on studies of isogenic populations, and they do not directly test the genetic aspects of evolutionary theories of aging. Furthermore, phenotypes such as progeny production and life span represent the outcomes of complex interactions between genetic and environmental factors (Reznick 1985). Strong environmental influences can, in principle, mask genetic relationships among traits, so that the resulting phenotypic correlations are more positive than the underlying genetics dictate (Lande 1982; Reznick 1985). Therefore, the results of our studies do not directly test the genetic basis of evolutionary theories of aging.

The results suggest that a common mechanism or pathway regulates progeny production, reproductive aging, and somatic aging in individual *C. elegans* hermaphrodites. The novel biomarkers of mated animals described here—cumulative progeny production through day 4, and daily progeny production on days 2, 4, and 5—will aid future analyses of the mechanisms regulating reproduction and aging.

## Supplementary Material

Supporting Information
