# Supplementary material for: Mated Progeny Production Is a Biomarker of Aging in Caenorhabditis elegans
Source: G3 (Bethesda). 2013 Oct 18;3(12):2219–32. doi: 10.1534/g3.113.008664 (PMC3852384; doi:10.1534/g3.113.008664)
Supplement: Supporting Information [file supp_3_12_2219__index.html]

Mated Progeny Production Is a Biomarker of Aging in Caenorhabditis elegans — Supporting Information 

# Mated Progeny Production Is a Biomarker of Aging in *Caenorhabditis elegans*

## Supporting Information for Pickett *et al.*, 2013

**Files in this Data Supplement:**

- File S1 - Outcome of every animal analyzed (.xlsx, 73 KB)
- Table S1 - 95% confidence intervals for Pearson correlation values for WT hermaphrodites (.xlsx, 10 KB)
- Table S2 - 95% confidence intervals for Pearson correlation values for cumulative progeny production (.xlsx, 10 KB)
- Table S3 - 95% confidence intervals for Pearson correlation values for daily progeny production (.xlsx, 10 KB)
- Table S4 - 95% confidence intervals for Pearson correlation values comparing early and late reproduction (.xlsx, 9 KB)
